# Mitf-Mdel, a novel melanocyte/melanoma-specific isoform of microphthalmia-associated transcription factor-M, as a candidate biomarker for melanoma

**DOI:** 10.1186/1741-7015-8-14

**Published:** 2010-02-17

**Authors:** Yixiang Wang, Soroosh Radfar, Suhu Liu, Adam I Riker, Hung T Khong

**Affiliations:** 1 Research Laboratory of Oral and Maxillofacial Surgery, Peking University School of Stomatology, Beijing, China; 2 Yale University School of Medicine, Department of Internal Medicine, Hematology section, 333 Cedar Street, PO Box 208021, New Haven, CT, USA; 3 Dana-Farber Cancer Institute, Boston, MA, USA; 4 Ochsner Cancer Institute, Department of Surgery 1514 Jefferson Highway, BH334 New Orleans, LA 70121, USA; 5 Mitchell Cancer Institute, University of South Alabama, 1660 Springhill Avenue, Mobile, AL 36604, USA

## Abstract

**Background:**

Melanoma incidence is on the rise and advanced melanoma carries an extremely poor prognosis. Treatment options, including chemotherapy and immunotherapy, are limited and offer low response rates and transient efficacy. Thus, identification of new melanocyte/melanoma antigens that serve as potential novel candidate biomarkers in melanoma is an important area for investigation.

**Methods:**

Full length MITF-M and its splice variant cDNA were cloned from human melanoma cell line 624 mel by reverse transcription polymerase chain reaction (RT-PCR). Expression was investigated using regular and quantitative RT-PCR in three normal melanocytes (NHEM), 31 melanoma cell lines, 21 frozen melanoma tissue samples, 18 blood samples (pheripheral blood mononuclear cell; PBMC) from healthy donors and 12 non-melanoma cancer cell lines, including three breast, five glioma, one sarcoma, two kidney and one ovarian cancer cell lines.

**Results:**

A novel splice variant of MITF-M, which we named MITF-Mdel, was identified. The predicted MITF-Mdel protein contains two in frame deletions, 56- and 6- amino acid deletions in exon 2 (from V32 to E87) and exon 6 (from A187 to T192), respectively. MITF-Mdel was widely expressed in melanocytes, melanoma cell lines and tissues, but almost undetectable in non-melanoma cell lines or PBMC from healthy donors. Both isoforms were expressed significantly higher in melanoma tissues than in cell lines. Two of 31 melanoma cell lines expressed only one isoform or the other.

**Conclusion:**

MITF-Mdel, a novel melanocyte/melanoma-specific isoform of MITF-M, may serve as a potential candidate biomarker for diagnostic and follow-up purposes in melanoma.

## Background

The microphthalmia-associated transcription factor (MITF) is a member of the basic helix-loop-helix leucine zipper transcription factor family, which plays a central role in the differentiation of neural crest-derived melanocytes, optic cup-derived retinal pigment epithelial cells, bone marrow-derived mast cells and osteoclasts and natural killer cells [1-5]. Mutations in MITF are associated with auditory-pigmentary syndromes such as Waardenburg syndrome [6] and Tietz syndrome [7,8] and result in hearing loss and depigmentation of the hair and skin.

The MITF gene is expressed in different isoforms that are under the control of distinct promoters. At least eight isoforms of human MITF with different N-termini are known, MITF-A[9], MITF-B[10], MITF-C[11], MITF-D[12], MITF-E[13], MITF-H[14], MITF-J [15] and MITF-M[16], and are derived from alternative splicing of a unique first exon. They all share the common downstream exons from 2 to 9. All isoforms share important functional domains including the transactivation domain, basic domain, and helix-loop-helix and leucine-zipper domain (b-HLH-LZ). Distinct isoforms may possess cell-specific functions. For example, MITF-M is exclusively expressed in melanocyte/melanoma cells and serves as the master gene for melanocyte development, survival and differentiation.

Several studies provide evidence that MITF serves as an oncogene in human melanoma. MITF amplification was found in 15-20% of metastatic melanoma, and is associated with decreased 5-year survival [17]. In addition, transformation of immortalized human melanocytes occurred through the cooperation of MITF and activated BRAF (*V600E*) [17].

The diagnosis of metastatic melanoma most often relies on S100 and HMB-45 melanoma biomarkers [18]. However, S100 is highly sensitive but not very specific as it also stains other nonmelanoma cancers. In contrast, HMB-45 is highly specific for melanoma but not very sensitive as it may miss a significant of melanoma cases. Therefore, the combination of both S100 and HMB-45 is often used to improve their diagnostic utility [18]. MITF has been shown to be superior to the S100 and HMB-45 combination in both sensitivity and specificity in the diagnosis of melanoma [18].

One of the most difficult decisions in the treatment of melanoma is whether a particular patient needs adjuvant therapy. Current approaches to adjuvant therapy in melanoma, including the use of high dose interferon-α, are associated with significant toxicity but with modest benefits. Therefore, it is important to have ways of identifying which patients are at higher risk of relapse and, therefore, may benefit from adjuvant therapy. One of the strategies that have been widely studied is the detection of circulating tumour cells, using a variety of molecular biomarkers. The most commonly used markers are the melanoma/melanocyte tissue-differentiation antigens, including tyrosinase and melanoma-associated antigen recognized by T cells (MART-1). However, the lower than expected frequency of detection of circulating tumor cells using these assays may limit their clinical utility [19]. Unlike other biomarkers in melanoma, MITF is expressed at various levels in almost all melanoma specimens [18,20]. This is most probably due to its essential function in the survival of the melanocyte linage [18,20]. In addition, MITF detection after treatment was a significant independent prognostic factor for relapse-free and overall survival [21]. Therefore, MITF or MITF isoforms have the potential of being an important biomarker for melanoma.

Here we report a novel splicing variant of MITF-M, which we named MITF-Mdel. This variant was cloned from the human melanoma cell line 624 mel. Compared with wild-type MITF-M, MITF-Mdel has two in-frame deletions. The 56-amino acid deletion from V32 to E87 in exon 2 has not been previously reported in human. The 6-amino acid deletion, ACIFPT, from A187 to T192 of MITF-M in exon 6 has been previously reported in MITF-A, -D and -H isoforms [1,4,5]. We found that MITF-Mdel was widely expressed in normal human melanocytes and melanoma cell lines as well as primary melanoma tissues, but was almost undetectable in non-melanoma cancer cell lines and pheriperal blood mononuclear cell (PBMC) from healthy donors. Our finding showed that MITF-Mdel expression is melanocyte/melanoma specific and thus potentially a valuable candidate biomarker for melanoma.

## Methods

### Cancer cell lines

All cell lines, including 31 melanoma, five glioma, four breast, two colon, one ovarian, one sarcoma, and one kidney cancer cell lines, in this study were grown in RPMI 1640 medium containing 10% heat-inactivated fetal calf serum and 100 units/ml penicillin-streptomycin Sigma-Aldrich (MO, USA). Normal human melanocytes (NHEM) were grown in Melanocyte Cell Basal Medium-4 with growth supplements (Clonetics MGM-4™ BulletKit (CC-3249), Cambrex, NJ, USA). The cells were harvested for RNA isolation when they were approximately 70-80% confluent and in healthy condition.

### Blood and frozen melanoma tissue samples

PBMC from 20 healthy donors and frozen primary melanoma tissue samples were used in this study. PBMC was isolated from normal donors' buffy coats using a standard density gradient centrifugation method (Ficoll, Invitrogen, CA, USA). Blood samples and 21 frozen melanoma tissue samples were used for RNA isolation using an RNA extraction kit (RNeasy Total RNA, Qiagen, CA, USA), following the manufacture's instructions. Buffy coats from anonymous health donors were obtained from the regional American Red Cross blood bank. All melanoma tissue samples were cryopreserved within 10 min of surgical excision in liquid nitrogen and stored within the Tissue Procurement Laboratory of the Moffitt Cancer Center as described previously [22].

### Cloning of Mitf-M isoforms

Total RNA was isolated from human melanoma cell line 624 mel and treated with RNase-free DNase I for 30 min. RT was performed simultaneously with oligo (dT) with first strand cDNA synthesis kit (Amersham Biosciences, NJ, USA). MITF-M primer design was based on published sequence of human MITF mRNA [17]. The full-length human MITF-M cDNA, which was isolated using the forward primer 5'-gcagatctatgctggaaatgctagaatataat-3' and reverse primer 5'-gaattcacaagtgtgctccg-3', was cloned into pCDNA3.1/V5-his topo vector (Invitrogen, CA, USA). After restriction enzyme digestion screening, two types of human MITF-M constructs were identified based on agarose gel analysis.

### DNA sequencing

Sequencing of the isolated human MITF-M clones was performed by the Biotechnological Laboratory Core Facility at Northwestern University. Searches for sequence homology were performed with the GeneBank database using the BLAST algorithm.

### Expression analysis by RT-PCR and quantitative PCR (qPCR)

Total RNA was extracted from the three NHEMs, 31 melanoma cell lines, 21 frozen melanoma tissue samples (Frtu), 18 blood samples (PBMC) from healthy donors, and 12 non-melanoma cancer cell lines, including three breast, five glioma, one sarcoma, two kidney and one ovarian cancer cell lines using an RNA extraction kit (RNeasy Total RNA, Qiagen, CA, USA) according to the instructions. Total RNA was treated with DNase I (Promega, WI, USA) to avoid residual genomic DNA contamination. First-strand cDNA was synthesized using a First-Strand Synthesis system (ABgene, Epsom, UK) for regular RT-PCR reaction. Wild type MITF-M was amplified using primer pairs 5'-ttatagtaccttctctttgccagtcc-3' (human MITF-M specific forward primer) and 5'-cttataaaatccctctttttcacagttgga-3' (reverse). Deletion isoform, MITF-Mdel, was amplified using the same MITF-M specific forward primer and the MITF-Mdel specific reverse primer 5'-cttataaaatccctgccgttgg-3'. The cDNA for qPCR was obtained using high capacity cDNA archive kit (Applied Biosystems, CA, USA) according to the instructions. qRT-PCR was performed using the Bio-Rad iQ5 real time PCR machine coupled with SYBR Green chemistry (Applied Biosystems, CA, USA). The primers are listed in Table 1. All PCR reactions were in 25 μL of total volume containing 12.5 μL of SYBR green PCR master mix, 40 ng cDNA, 300 nM of each primer. All amplifications were done in triplicate for each sample and repeated once. The thermal cycling was 10 min at 95°C, followed by 40 cycles at 95°C for 15 s, at indicated annealing temperature for each gene in Table 1 for 20 s, and at 72°C for 30 s. The specificity of amplification was monitored using the dissociation curve of the amplified product. Relative expression of the target genes was calculated using delta Ct method, and 624 mel was used as a normalization control.

**Table 1:** Primer sequences for mRNA analysis by real-time polymerase chain reaction.

Gene	Primers (human)	Amplicon	Tm
GADPH	F: 5'-cgagatccctccaaaatcaa-3';	170	60
	R: 5'-ttcacacccatgacgaacat-3'		
MITF-M	F: 5'-ttatagtaccttctctttgccagtcc-3'	146	52
	R: 5'-gtttatttgctaaagtggtagaaaggtact-3'		
MITF-Mdel	F: 5'-ttatagtaccttctctttgccagtcc-3';	120	52
	R: 5'-cttataaaatccctgccgttgg-3'		

### Statistical analysis

Quantitative PCR data were expressed as median. The Student *t*-test was used in the analysis. Frequency rate was analysed by chi square test. *P *< 0.05 was considered statistically significant.

## Results

### Identification of a novel isoform of MITF-M, MITF-Mdel

**Figure 1:**
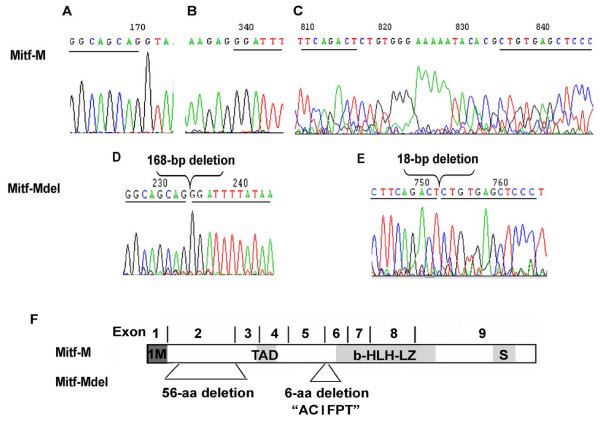
**Mutation analysis**. (A-E) Sequence traces showing the splicing site mutation of human microphthalmia-associated transcription factor (MITF)-M. (A&B) Sequence of exon 2 of wild-type MITF-M using the forward primer. (C) Sequence of exon 6 of wild-type MITF-M using the reverse primer. (D) Sequence of exon 2 of the MITF-Mdel using the forward primer. (E) Sequence of exon 6 of the MITF-Mdel using the reverse primer. The nucleotides between the underlined nucleotides indicate the deletion regions. (F) Schematic representation of the structures of MITF-M and MITF-Mdel. The MITF-Mdel isoform has a 168-bp in-frame deletion in exon 2 and an 18-bp in-frame deletion in exon 6, but still has the transcriptional activation domain, the b-HLH-LZ domain and the serine-rich region (S).

**Figure 2:**
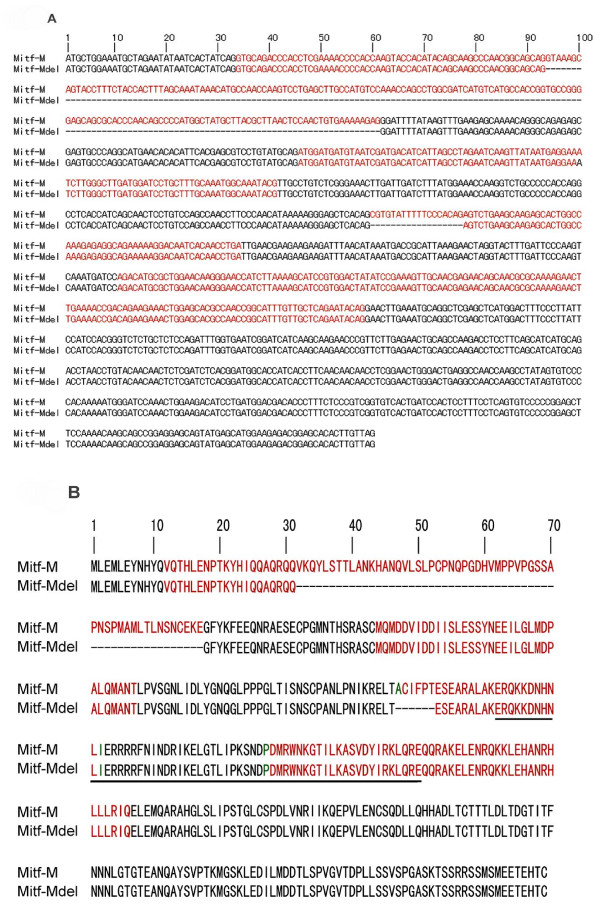
**cDNA and amino-acid sequences of human microphthalmia-associated transcription factor (MITF)-M and MITF-Mdel**. (A) Alignment of mRNA sequences of MITF-M and MITF-Mdel. MITF-Mdel contains 168-bp and 18-bp in-frame deletions at mRNA level. (B) Alignment of predicted protein sequences of MITF-M and MITF-Mdel. MITF-Mdel is predicted to encompass 357 amino acids. The underlined portion marks the basic-helix-loop-helix domain. Compared with MITF-M, MITF-Mdel has deletions of 56-amino acid (from V32 to E87) in exon 2 and 6-amino acid (from A187 to T192) in exon 6. Dash lines represent deletions. Red highlighting indicates alternate exons. Green highlighting indicates amino acids encoded across a splice junction.

The MITF-M cDNA fragment was obtained by RT-PCR of the mRNA from human melanoma cell line 624 mel and cloned into the pCDNA3.1/V5-his topo vector. After digestion by EcoRI and BglII, two distinctly different patterns were identified on agarose gel. DNA sequencing revealed that one was wild type MITF-M, and the other was a novel isoform of MITF-M, which we named MITF-Mdel. Compared with wild type MITF-M, MITF-Mdel contained two in-frame deletions (Figure 1). One deletion was in exon 2, with 168 bp (from 94G to 261G in the coding region of MITF-M) at mRNA level or 56- amino acid (from V32 to E87) at protein level, which had not been previously reported in human. The other deletion, with 18 bp (from 560C to 577G in the coding region of MITF-M) at mRNA level or 6-amino acid (from A187 to T192) at protein level, was located in exon 6, upstream from the basic region, and had previously been described [1,4,5]. However, the MITF-Mdel isoform still contained an entire basic helix-loop-helix leucine zipper domain, an important functional domain in MITF proteins (Figure 2a and 2b). MITF-Mdel sequence was deposited in GenBank (Accession No. GU355676).

### Expression profiles of MITF-M and MITF-Mdel

**Figure 3:**
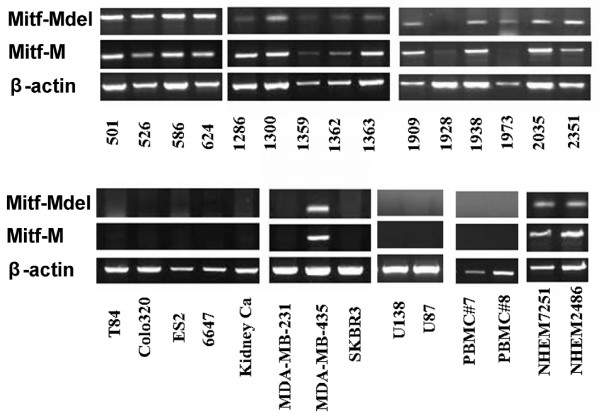
**Detection of microphthalmia-associated transcription factor (MITF)-Mdel and MITF-M in melanoma cell lines and other carcinoma cell lines by reverse transcriptase polymerase chain reaction (representative results)**. MITF-M and MITF-Mdel have similar expression profiles. Similar to MITF-M, MITF-Mdel was found to be expressed only in normal human melanocytes, melanoma cell lines, and fresh melanoma tissues. Both isoforms were also expressed in MDA-MB-435, a cell line originally thought to be a breast cancer cell line, but has been identified as a melanoma cell line. Most melanoma expressed both MITF-M isoforms while two melanoma cell lines expressed only one or the other. Melanoma cell lines (*n *= 31; only 16 shown): from 501 to 2351, and MDA-MB-435. Breast carcinoma cell lines (*n *= 3; only two shown): MDA-MB-231 and SKBR3. Colon cancer cell lines (*n *= 2): T84 and Colo320. Ovarian cancer cell line (*n *= 1): ES2. Glioma cell lines (*n *= 5; only two shown): U138 and U87. PBMC No.7 and No.8 from healthy donors. Normal human melanocytes (NHEM) 7251 and NHEM2486: normal human melanocytes.

The expression profiles of the two MITF-M isoforms in various tumor cell lines, normal human melanocytes, frozen fresh melanoma tissue samples and normal PBMC samples were analysed by RT-PCR and quantitative RT-PCR. As seen in Table 2, three of four melanocyte samples (75%), 29 of 31 melanoma cell lines (93.5%) and 21 of 21 frozen fresh melanoma tissue samples (100%) were positive for MITF-Mdel. Similar results were also obtained for wild type MITF-M. In addition, 30 of 31 melanoma cell lines (96.8%) were positive for either isoform. MITF-M and MITF-Mdel had similar patterns of expression. In this RT-PCR assay, they were found to be expressed only in normal human melanocytes, melanoma cell lines, and fresh melanoma tissues. Most expressed both MITF-M isoforms, while 2 of 31 melanoma cell lines expressed one or the other. Neither normal PBMC samples nor non-melanoma cancer cell lines analysed, including five glioma (LN229, A172, U138, U87 and U373), three breast (MDA-MB-231, SKBR3 and MCF-7), one ovarian (ES2), one sarcoma (6647), one kidney and two colon (Colo320 and T84) cancer cell lines were found to express either isoform of MITF-M (Figure 3).

**Table 2:** Detection of microphthalmia-associated transcription factor (MITF)-M and MITF-Mdel mRNA expression in normal human melanocytes (NHEM) and melanoma by reverse transcriptase polymerase chain reaction.

	MITF-M (+)		MITF-Mdel (+)		MITF-M or MITF-Mdel (+)	
	
	**No**.	%	**No**.	%	**No**.	%
NHEM	3/4	75%	3/4	75%	3/4	75%
Melanoma cell lines	29/31	93.5%	29/31	93.5%	30/31	96.8%
Melanoma tissue samples	21/21	100%	21/21	100%	21/21	100%

**Figure 4:**
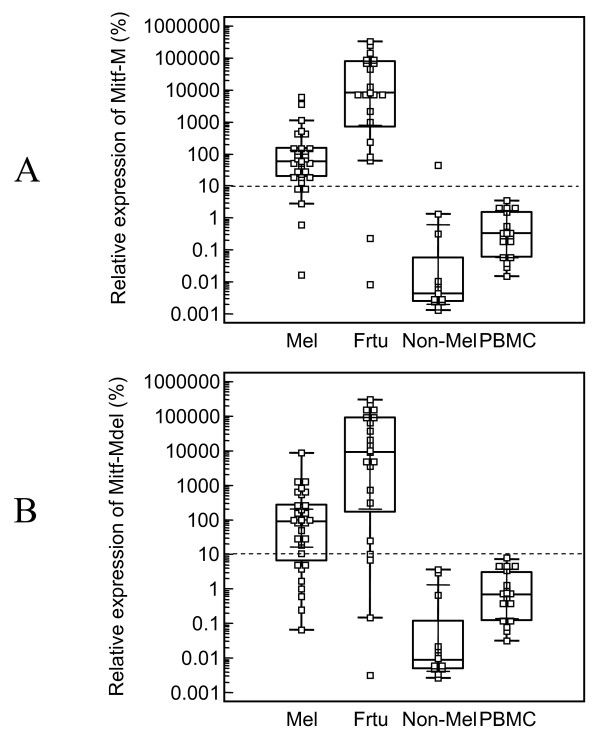
**Relative expression of microphthalmia-associated transcription factor (MITF)-M (A) and MITF-Mdel (B) in 31 melanoma cell lines, 21 frozen melanoma tissue samples, 18 PBMCs from healthy donors, and 12 non-melanoma cell lines, including three breast, five glioma, one sarcoma, two kidney and one ovarian cancer cell lines**. Data was calculated using ΔΔCt method. Melanoma cell line 624 mel was used as normalization control and its MITF-M and MITF-Mdel relative expression levels were set as 100%. Bars indicated median of expression levels of MITF-M or MITF-Mdel mRNA in each group.

RT-qPCR showed similar results as regular RT-PCR (Figure 4a and 4b). 624 mel cell line was used as a reference to normalize measurements from real-time PCR. The mRNA expression level of MITF-M and MITF-Mdel in 624 mel was referenced as 100%. Based on the variation of mRNA status among samples, relative mRNA expression level of MITF-M and MITF-Mdel were used to assess the cutoff point. The cutoff point was above the mean relative MITF-M or MITF-Mdel plus 5 × SD of normal PBMCs. We set the cutoff for MITF-M and MITF-Mdel positivity at 10% of the mRNA expression level of those in 624 mel cell line.

The cutoff line for MITF-M was above the MITF-M levels of all normal PBMCs and all but one of the non-melanoma cell lines, but above the MITF-M levels of 25 of 30 (83.3%) melanoma cell lines, and 19 of 21 (90.5%) frozen melanoma tissue samples. While the cutoff line for MITF-Mdel was above MITF-Mdel levels of all normal PBMCs and all of the non-melanoma cell lines, but above the MITF-Mdel levels of 22 of 30 (73.3%) melanoma cell lines, and 18 of 21 (85.7%) frozen melanoma tissue samples. Specimens with higher relative MITF-M or MITF-Mdel expression level than the cutoff point were considered as positive for MITF-M or MITF-Mdel. Both MITF-Mdel and MITF-M levels of expression in frozen melanoma tissue samples were significantly higher than those in melanoma cell lines (*P *= 0.0001), PBMCs from normal donors (*P *< 0.0001) and all of the non-melanoma cell lines (*P *< 0.0001).

## Discussion

In this report, MITF-Mdel, a novel melanocyte/melanoma-specific isoform of MITF-M, was cloned from the human melanoma cell line 624 mel. The predicted MITF-Mdel protein contains two in frame deletions, 56- and 6- amino acid deletions in exon 2 (from V32 to E87) and exon 6 (from A187 to T192), respectively. The former deletion had not been previously reported. MITF-Mdel was widely expressed in melanocytes, melanoma cell lines and tissues, but almost undetectable in non-melanoma cell lines or PBMC from healthy donors. Both isoforms were expressed significantly higher in melanoma tissues than in cell lines. Two of 31 melanoma cell lines expressed only one isoform or the other. As seen in Figure 4, the medians of relative expression of MITF-M and MITF-Mdel by qRT-PCR are comparable. Therefore, MITF-Mdel seems to be a common isoform in melanoma. At this time, we do not know if there are differences in the relative amounts of each isoform in melanocytes versus primary melanoma versus metastatic melanoma. By correlating the differential expression of these MITF-M isoforms in primary and metastatic melanoma tissues with progression free survival and overall survival in future studies, we may be able to validate the utility of MITF-Mdel as a biomarker.

We analyzed the sequence of MITF-Mdel and the 168-bp deletion in exon 2 and found a cryptic splice donor site 'GTAAA' [23] at the beginning of the 168-bp deletion. The last nucleotide of the 168-bp deletion is at the exon-intron boundary and can be considered as the acceptor site. This may provide a mechanistic explanation for the generation of MITF-Mdel splice variant. In addition, human MITF-M mRNA with an 18-bp deletion has been shown to result from alternative splicing using two acceptor sites located at the 5'end of exon 6 [24].

Similar to MITF-M, MITF-Mdel is driven by the M promoter and contains the transcriptional activation domain, the b-HLH-LZ domain and the serine-rich region. Whether the 168-bp deletion in exon 2 of MITF-Mdel has any phenotypic or functional consequence is not known at this time. Since serine 73 (Ser73), which is phosphorylated by mitogen activated protein kinase cascade, is located in exon 2B of the MITF gene, the newly discovered deletion from V32 to E87 effectively removes this phosphorylation site in MITF. Phosphorylation does not seem to affect accumulation of MITF in the nucleus [25]. However, mutation at Ser73 has been shown to reduce MITF's transcriptional activity on the tyrosinase promoter [25,26]. Other recent studies did not support the essential role of Ser73 or of exon 2 in the function of MITF. The serine to alanine mutation (Ser73Ala) did not alter the phenotype of the mutant mice [27]. In addition, deletion of exon 2 did not affect the function of MITF [28].

Melanoma cell line 1973 carries only the MITF-Mdel isoform and not the wild-type MITF; whereas melanoma cell line 624 carries both isoforms (Figure 3). From our previous study, it seems that MITF is still functional in 1973 mel. Downstream target genes for MITF, including tyrosinase, tyrosinase-related protein (TRP)-2, gp100, and MART-1, were expressed at comparable levels as those expressed in 624 mel [29].

Since adjuvant treatments for melanoma may have limited benefits but much potential toxicity, the ability to identify patients at high risk for recurrence is essential in the development of an effective adjuvant therapy. A highly sensitive RT-PCR and real-time quantitative RT-PCR offer a platform for monitoring circulating melanoma cells to potentially predict melanoma prognosis and identify high-risk patients for further treatment.

Tyrosinase, MART-1, and gp100 are melanocyte/melanoma-differentiation antigens that are frequently expressed in melanoma cells and not in non-melanoma tumours. In one study, circulating tyrosinase and MART-1 mRNA was detected in only 77% and 54%, respectively in these patients [30]. MITF-M is another melanocyte/melanoma-specific marker that has been used to detect circulating tumour cells. In a recent study, MITF expression by quantitative RT-PCR was almost undetectable in PBMC of healthy blood donors, as in our study. However, it was detected in 86% of melanoma tissue samples. The rate of circulating MITF detection was higher with increasing melanoma stages. MITF detection after treatment was a significant independent prognostic factor for relapse-free and overall survival [21].

## Conclusions

The novel isoform MITF-MDel was widely expressed in melanocytes, melanoma cell lines and tissues, but almost undetectable in non-melanoma cell lines or PBMC from healthy donors, and may serve as a potential candidate biomarker for diagnostic and follow-up purposes in melanoma.

## Abbreviations

b-HLH-LZ: helix-loop-helix and leucine-zipper; MART-1: melanoma-associated antigen recognized by T cells; MITF: microphthalmia-associated transcription factor; NHEM: normal human melanocytes; PBMC: pheripheral blood mononuclear cell; qPCR: quantitative PCR; RT-PCR: reverse transcriptase polymerase chain reaction.

## Competing interests

The authors declare that they have no competing interests.

## Authors' contributions

YW cloned the MITF-Mdel gene, investigated its expression profiling via RT-PCR and qPCR and drafted the manuscript. SR isolated PMBC from healthy donors' blood. SL carried out the RNA extraction. AR contributed melanoma tissue samples. HK participated in the design of the study and interpretation of the data, and helped to draft the manuscript. All authors read and approved the final manuscript.

## Pre-publication history

The pre-publication history for this paper can be accessed here:

http://www.biomedcentral.com/1741-7015/8/14/prepub
